# Tirzepatide, a dual GIP/GLP-1 receptor agonist, alleviates metabolic dysfunction-associated steatotic liver disease by reducing the expression of CD36 and OBP2A

**DOI:** 10.1016/j.gendis.2025.101761

**Published:** 2025-07-03

**Authors:** Yun Li, Wencong Sun, Hong Liu, Xiong Z. Ruan

**Affiliations:** a Centre for Lipid Research & Chongqing Key Laboratory of Metabolism on Lipid and Glucose, The Second Affiliated Hospital, Chongqing Medical University, Chongqing 400016, China; b John Moorhead Laboratory, Centre for Nephrology, University College London, Rowland Hill Street, London, NW3 2PF, UK

**Keywords:** Adipose triglyceride lipase, Fatty acid translocase, Metabolicdysfunction-associated steatotic liver disease, Odorant binding protein 2a, Tirzepatide

## Abstract

Metabolic dysfunction-associated steatotic liver disease (MASLD) is defined by excessive hepatic lipid accumulation. This study evaluated the therapeutic effects and molecular mechanisms of tirzepatide, a dual GIP and GLP-1 receptor agonist, in treating hepatic steatosis. Eight-week-old C57BL/6J mice were fed either a high-fat diet or a high-fat, high-fructose, and high-cholesterol diet for 12 weeks to induce MASLD. From week 8, some mice received weekly intraperitoneal tirzepatide injections for four weeks. Tirzepatide significantly reduced body and liver weight gain. Histological analysis confirmed decreased hepatic vacuolation and lipid deposition. The drug also lowered serum glucose levels and reduced liver triglyceride and cholesterol content without causing liver injury. Transcriptome analysis showed that tirzepatide downregulated mitochondrial oxidative phosphorylation pathways. It also decreased hepatic expression of CD36 and odorant-binding protein 2A, both involved in lipid uptake. Importantly, tirzepatide did not significantly alter other major liver metabolic pathways. In adipose tissue, it reduced CD36 and odorant-binding protein 2A expression and upregulated adipose triglyceride lipase, suggesting enhanced lipolysis. However, it had no effect on CD36 levels in skeletal muscle. These results suggest that tirzepatide may be an effective treatment for MASLD by reducing liver fat accumulation and modulating lipid metabolism in extrahepatic tissues.

## Introduction

Metabolic dysfunction-associated steatohepatitis (MASLD) is a liver disease closely related to metabolic syndrome characterized by the abnormal accumulation of lipids within hepatocytes, inflammation, and fibrosis.^1^^,^^2^ The pathogenesis of MASLD is complex, involving the interaction of multiple factors, including genetic predisposition, environmental influences, and lifestyle choices. In recent years, with the rise in obesity and type 2 diabetes mellitus, MASLD has become an increasingly serious public health issue globally.^3^^,^^4^ To find effective therapeutic strategies, researchers are exploring new drugs that can improve hepatic lipid metabolism, alleviate liver burden, and reverse or halt disease progression.

Tirzepatide, a novel dual agonist for both glucagon-like peptide-1 (GLP-1) and glucose-dependent insulinotropic polypeptide (GIP) receptors,^5^ has demonstrated excellent glucose-lowering effects in preclinical studies and has been used to improve glycemic control in patients with type 2 diabetes.^6^, ^7^, ^8^, ^9^, ^10^ Tirzepatide is also under review for chronic weight management. Among persons with moderate-to-severe obstructive sleep apnea and obesity, tirzepatide reduced the apnea–hypopnea index, body weight, hypoxic burden, high-sensitivity C-reactive protein concentration, and systolic blood pressure and improved sleep-related patient-reported outcomes.^11^ In the SURMOUNT clinical trial programs, once-weekly tirzepatide has demonstrated sustained reductions in body weight among adults with obesity or overweight, accompanied by improvements in cardiometabolic risk factors.^12^^,^^13^ In addition to its superior efficacy in the management of type 2 diabetes and obesity, preclinical studies suggest that tirzepatide may also have potential protective effects on biomarkers of nonalcoholic steatohepatitis in patients with type 2 diabetes and that treatment with tirzepatide for 52 weeks was effective with respect to the resolution of MASH without worsening fibrosis.^14^, ^15^, ^16^, ^17^ The dual-action mechanism of tirzepatide makes it a candidate for treating MASLD; however, the underlying molecular mechanisms by which tirzepatide alleviates MASLD are largely unknown.

MASLD involves multiple pathways of liver metabolic abnormalities, including lipid metabolism disorders, insulin signaling defects, inflammatory responses, and enhanced oxidative stress, which are interrelated and collectively promote the occurrence and development of MASLD.^18^^,^^19^ This study aims to systematically evaluate the impact of tirzepatide on hepatic pathological changes, triglycerides, and cholesterol, as well as the levels of liver enzymes and blood glucose in MASLD, particularly to systematically evaluate key metabolic pathways and protein expression using gene screening and molecular biology techniques. Additionally, we examined the impact of tirzepatide on the expression of metabolic proteins in adipose tissue and muscle tissue to further elucidate its mechanisms in improving systemic metabolic status in MASLD. A thorough examination of the molecular mechanisms of tirzepatide in MAFLD may be essential to provide scientific evidence for the application of tirzepatide in MASLD treatment and establish a foundation for the development of targeted therapeutic strategies for MASLD.

## Materials and methods

### Reagents and antibodies

Tirzepatide was purchased from Selleck (Shanghai, China). Triglyceride, total cholesterol, alanine aminotransferase (ALT) and aspartate aminotransferase (AST) assay kits were purchased from Zhongsheng Beikong Biotechnology (Beijing, China). HCS LipidTOX™ Deep Red neutral lipid stain (Cat. H34477) and a Nile Red Staining Kit (Cat. C2051S) were purchased from Invitrogen and Beyotime, respectively. Optic atrophy protein 1 (OPA1) antibody (1:1000, cat. ET1705-9), COX IV antibody (1:2000, cat. ET1701-63), cytochrome C antibody (1:3000, cat. ET1610-16) and adipose triglyceride lipase antibody (1:1000, cat. HA721951) was obtained from HUABIO Biotechnology (Hangzhou, China). CD36 antibody (1:2000, cat. NB400-144) was obtained from Novus Biologicals (Colorado, USA). Olfactory binding protein 2A (OBP2A) antibody (cat. bs-21128R) was obtained from Bioss (Beijing, China). A human oxidative phosphorylation immunoblotting kit (Cat. PK30006) was obtained from Proteintech (Wuhan, China). chREBP antibody (1:1000, cat. ab92809), rabbit monoclonal to AMPK alpha 1 (phospho T183) and AMPK alpha 2 (phospho T172) (1:1000, cat. ab133448), rabbit monoclonal to AMPK alpha 1 (1:1000, cat. ab32047) and rabbit monoclonal to AMPK alpha 2 (1:1000, cat. ab214425) were purchased from Abcam (Cambridge, MA). The CD36 overexpression lentivirus, OBP2A overexpression plasmid, and siRNA targeting OBP2A were purchased from OBiO technology (Shanghai, China). The siRNA targeting CD36 was obtained from Sangon Biotech (Shanghai, China).

### Animal models and treatments

Eight-week-old male C57BL/6J mice were purchased from Ensiweier (China) and housed under specific pathogen-free (SPF) conditions. The mice were randomly divided into multiple groups, with at least five animals per group. Besides the normal chow diet (NCD) group, other mice were fed either a high-fat diet (HFD) (Research Diets, D12492) or a high-fat, high-fructose, and high-cholesterol (HFFC) diet (Research Diets, D09100310) for 12 weeks to induce MASLD. One group of mice that were fed with HFD or HFFC diet began to receive weekly intraperitoneal injections of tirzepatide (0.25 mg/kg body weight) for 4 consecutive weeks starting from the eighth week, while the remaining mice fed an HFD or HFFC diet received an equivalent volume of saline as a control. Body weight was recorded weekly throughout the experiment. At 12.5 weeks, the mice were euthanized, and the liver weights were immediately measured and recorded. All animal experimental protocols were approved by the Institutional Animal Care and Use Committee at the Second Affiliated Hospital of Chongqing Medical University.

### Liver histological analysis

Livers samples were fixed in 10% phosphate-buffered formalin acetate at 4 °C overnight and embedded in paraffin wax. Paraffin sections (5 μm) were cut and mounted on glass slides for hematoxylin‒eosin (HE) staining. Immunohistochemistry of liver sections was performed. Livers embedded in optimum cutting temperature compound (Tissue-Tek, Laborimpex) were used for Oil Red O staining for the assessment of hepatic steatosis according to the manufacturer’s instructions (Beyotime).

### Transcriptome sequencing, differential expression analysis, and functional enrichment

Total RNA was extracted from the liver tissue using the TRIzol® Reagent according to the manufacturer’s instructions. Then the RNA quality was determined and quantified. Only high-quality RNA samples (OD_260_/_280_ = 1.8–2.2, OD_260_/_230_ ≥ 2.0, RQN ≥6.5, 28S:18S ≥ 1.0, >1 μg) were used to construct a sequencing library. RNA purification, reverse transcription, library construction and sequencing were performed at Shanghai Majorbio Bio-pharm Biotechnology Co., Ltd. (Shanghai, China) according to the manufacturer’s instructions. The RNA-seq transcriptome library was prepared following Illumina® Stranded mRNA Prep, Ligation (San Diego, CA) using 1 μg of total RNA. After quality control, the raw data were aligned to a reference genome, and gene expression levels were calculated. GO functional enrichment and KEGG pathway analyses were carried out via Goatools and Python SciPy software, respectively.

### RNA isolation and quantitative PCR

Liver tissues or cells were lysed in TRIzol® Reagent to extract total RNA according to the manufacturer’s protocol. Total RNA was reverse-transcribed into cDNA using a reverse transcription kit (HY–K0511, MCE) according to the manufacturer’s instructions. Quantitative PCR reactions were carried out using SYBR Green qPCR master mix (HY–K0501, MCE), with β-actin used as the reference gene for relative quantification of target mRNA expression levels. The following primers were used: for mouse Actb, TCTTTGCAGCTCCTTCGT (F) and GACCCATTCCCACCATC (R); for mouse Gsta2, AGCCTTCTGACCCCTTTC (F) and CATCAATGCAGCCACACTA (R); for mouse Slc26a2, TACCTCTCCGACGCCTT (F) and AATGACTGAGCCGACACC (R); for mouse Nox4, GCTCATTTCCCACAGACC (F) and CGCACAATAAAGGCACAA (R); for mouse Hif1a, TGAACCCATTCCTCATCC (F) and CGGCCCAAAAGTTCTTC (R); for mouse Atp5mc2, GTTTGTCTGTGTCCCGTGT (F) and CTGTTGAGTGGCAGCGT (R); for mouse cytochrome c, somatic (Cycs), CGTTCGTGGTGTTGACC (F) and CTTATGCTTGCCTCCCTTT (R); for mouse Opa1, GACAAAGGCATCCACCAC (F) and CTCCAACCACAACAACCC (R); for mouse Socs2, GCTGGACCGACTAACCTG (F) and CCGTTTATCCTTGCACATC (R); for mouse Dusp1, CTGTTGTTGGATTGTCGCT (F) and GTTGGGCACGATATGCTC (R); for mouse Aldoc, AAGCAGGGATCAGAGTGG (F) and TGGAAGTGGGTCATAGCC (R); for mouse Tgtp1 TGATGTGGATTTGGTTTGC (F) and CCGATGTCCCTGTTTCC (R); for mouse Ubd, TGTGACCTCTGTGATCCCT (F) and CTTCCAGCTTCTTTCCGTT (R); for mouse OBP2A, ACCGCTTTTCATAACACGA (F) and GCCTCTGGATTTTCACCA (R); for mouse Cxcl9, TTCTCCCTCCCTCCCTT (F) and CTCTGTGCGCTGAAGATG (R); for mouse Cxcl10, TTTCTGCCTCATCCTGCT (F) and CCCTATGGCCCTCATTCT (R); for mouse CD36, AAC AGC AGC AAA ATC AAG GT (F) and GAC AGT GAA GGC TCA AAG ATG (R); for mouse Slc27a5, TAACGTCCCTGAGCAACC (F) and CCCACATTGCCCTCTGT (R); for mouse Slc27a4, CTTGGGGTCTGGTGGCT (F) and ACCGTCTTCCGCTCCTG (R); for mouse Slc27a1, CAGGAGTGGAGGGGAAAG (F) and AAGACGCAGGAAGATGGG (R); for mouse Plin1, CCTGGTGGCCTCTGTGT (F) and AGCTGTGAACTGGGTGGAC (R); for mouse adipose triglyceride lipase (ATGL), CCTATACTCTGCCGCTGGA (F) and TGCTACCCGTCTGCTCTTT (R); for mouse Ccl2, ACCTTTTCCACAACCACCT (F) and GCATCACAGTCCGAGTCA (R); for mouse Mgll, ACAGCCCTCGTTTGCCT (F) and GTGGAGTTCGCCTGGGT (R); for mouse Lipe, GCCTCATGGACCCTCTTCT (F) and ACGCCTAGTGCCTTCTGG (R); for mouse Scd1, GCCTCTTCGGGATTTTCT GTCATTCTGGAACGCCAT (R); for mouse Fasn, TCCAAGTGCTCGTGTCAAC (F) and TCTGGGGTCTGGTTCTCC (R); for mouse Acaca, AGTGCCCTCAATTCTGTCC (F) and GGTTCTCTGCTCCAAGTCC (R); for mouse Srebf2, CAAGTCAGCAGCCAAGGA (F) and CCACCGCTCTTTCTAGCAG (R).

### Western blotting

Protein samples were separated by SDS‒PAGE and transferred onto PVDF membranes. The membranes were blocked with 0.3% bovine serum albumin and incubated with primary antibodies overnight at 4 °C. The following day, after the samples were washed three times, they were incubated with the corresponding secondary antibodies. The protein bands were detected using chemiluminescent reagents, and ImageJ software was used for quantitative analysis.

### Immunohistochemistry

The tissue samples were fixed in 4% paraformaldehyde, embedded in paraffin, and cut into 5 μm sections. After dewaxing and antigen retrieval, the sections were treated with 3% hydrogen peroxide to block endogenous peroxidase activity. The sections were then blocked in 5% goat serum for 1 h and incubated overnight at 4 °C with diluted primary antibodies. The next day, the sections were incubated for 1 h at room temperature with HRP-conjugated secondary antibodies, followed by DAB staining and counterstaining with hematoxylin.

### Statistical analysis

The data are expressed as mean ± SD. Statistical significance was evaluated using the unpaired two-tailed Student’s *t*-test. Differences were considered significant at a *P* value < 0.05. Data analysis was performed using GraphPad Prism 10.0.

## Results

### Tirzepatide alleviates lipid accumulation in the livers of MASLD mice

**Figure 1:**
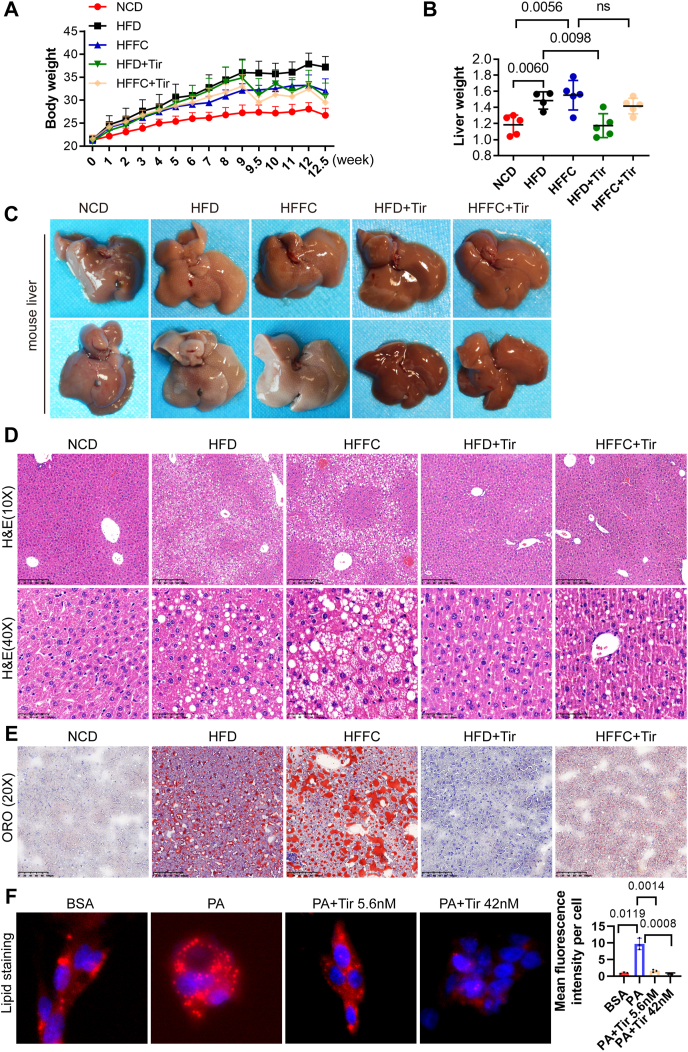
Administration of tirzepatide significantly attenuates hepatic lipid accumulation in MASLD mice. Effect of tirzepatide treatment on body weight **(A**) and liver weight **(B)** in mice with MASLD. Representative images of liver appearance **(C),** histological analysis via hematoxylin‒eosin (HE) staining **(D)**, and lipid accumulation assessment via Oil Red O staining **(E)** are shown. **(F)** HepG2 cells were incubated with 0.2% BSA, 0.2 mM PA, or 0.2 mM PA supplemented with 5.6 nm or 42 nm tirzepatide for 24 h. Cellular lipid accumulation was visualized by Nile Red staining, and the average lipid content per cell was quantified using ImageJ software. *n* = 5 for A–E and *n* = 3 for F. Data are presented as mean ± SD. ns, not significant; Tir, tirzepatide.

To determine whether tirzepatide can alleviate hepatic steatosis, 8-week-old C57BL/6J mice were fed with an HFD or HFFC for 12 weeks to establish a model of MASLD. From the 8th week of feeding, a subset of these mice was randomly assigned to receive weekly intraperitoneal injections of tirzepatide (0.25 mg/kg) for four consecutive weeks. Body weight was monitored throughout the study, revealing that both HFD and HFFC feeding significantly promoted sustained weight gain in mice, an effect that was attenuated by tirzepatide administration. At 12.5 weeks, upon sacrifice, the mice on the HFD or HFFC had significantly greater body weights than those on the NCD, while within the HFD or HFFC groups, the tirzepatide-treated mice weighed significantly less than their non-treated counterparts, indicating an evident weight-reducing effect of tirzepatide (Fig. 1A). The liver weights of the mice on the HFD or HFFC were also significantly greater than those on the NCD, but this increase was significantly mitigated in the tirzepatide-treated group (Fig. 1B). Macroscopic examination revealed that the livers of HFD- or HFFC-fed mice exhibited a pale appearance, consistent with lipid accumulation, whereas tirzepatide treatment markedly reduced this hepatic pallor (Fig. 1C). HE staining revealed marked vacuolation and hepatocyte enlargement in the livers of HFD- or HFFC-fed mice compared to those of the NCD group, and pathological changes that were significantly ameliorated by tirzepatide treatment (Fig. 1D). Consistent with these findings, Oil Red O staining demonstrated significant hepatic lipid accumulation in HFD- and HFFC-fed mice, which was markedly reduced following tirzepatide administration (Fig. 1E). *In vitro*, palmitic acid induced lipid accumulation in HepG2 cells, whereas tirzepatide attenuated this effect (Fig. 1F). Taken together, these findings suggest that tirzepatide effectively alleviates hepatic steatosis in MASLD.

### Tirzepatide decreases hepatic triglyceride, cholesterol, and serum glucose levels in MASLD

**Figure 2:**
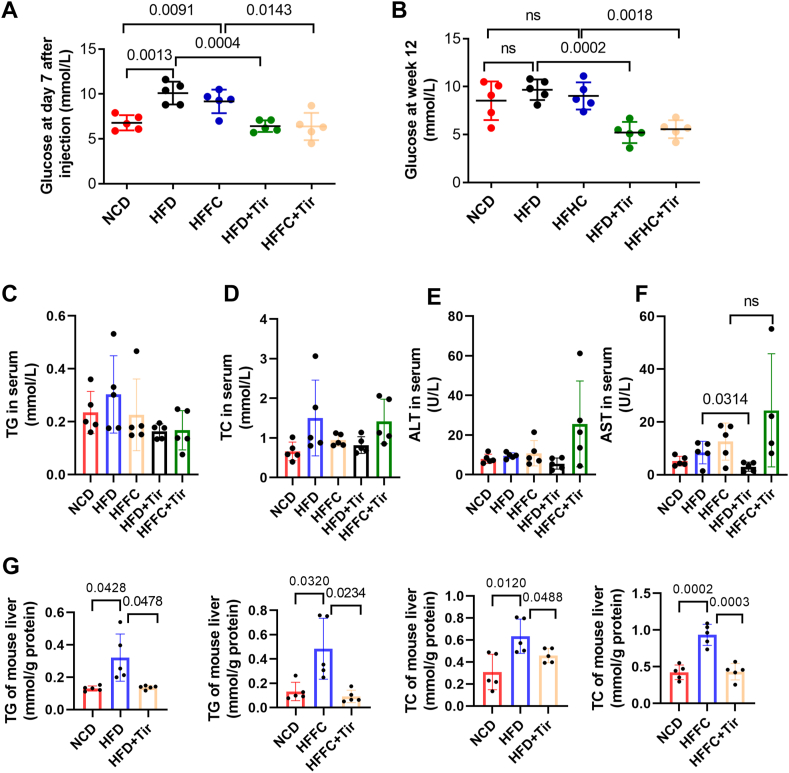
Tirzepatide decreases hepatic triglyceride, cholesterol, and serum glucose levels in MASLD. The mice were fed with NCD, HFD, or HFFC diets and received either tirzepatide injection or no treatment. Blood glucose levels were measured seven days after tirzepatide administration **(A)**, as well as at the time of sacrifice **(B)**. Serum TG **(C)**, TC **(D)**, ALT **(E)**, and AST **(F)** were measured in the five groups of mice. Hepatic TG and TC were measured in the five groups of mice **(G)**. *n* = 5. The data are presented as mean ± SD. TG, triglyceride; TC, total cholesterol; ALT, alanine aminotransferase; AST, aspartate aminotransferase.

Given that tirzepatide has been approved for glycemic control in patients with diabetes, we evaluated its glucose-lowering and lipid-regulating effects in a MASLD mouse model. As shown in Figure 2A, following seven days of tirzepatide administration, blood glucose levels measured via tail vein sampling were significantly lower in the treatment group than in the vehicle-injected controls. At the time of sacrifice, serum glucose levels remained significantly lower in the tirzepatide-treated group than in the HFD- or HFFC-fed groups (Fig. 2B), demonstrating its effective glucose-lowering activity in MASLD. Further analysis of serum lipids revealed that 12 weeks of HFD feeding did not significantly alter serum triglyceride or total cholesterol levels, and tirzepatide treatment did not induce significant reductions in these parameters (Fig. 2C, D). To investigate whether tirzepatide causes drug-induced liver injury, we measured ALT and AST levels. As shown in Figure 2E and F, tirzepatide reduced AST levels under HFD conditions without altering ALT levels or affecting AST levels in HFFC-fed mice, suggesting the absence of drug-induced liver injury. For hepatic lipids, we observed that HFD or HFFC feeding significantly increased liver triglyceride and total cholesterol levels, while tirzepatide treatment significantly reduced the accumulation of both triglycerides and total cholesterol (Fig. 2G). Taken together, tirzepatide decreases hepatic triglyceride, cholesterol, and serum glucose levels in MASLD.

### Mitochondrial oxidative phosphorylation pathways were down-regulated after tirzepatide treatment

**Figure 3:**
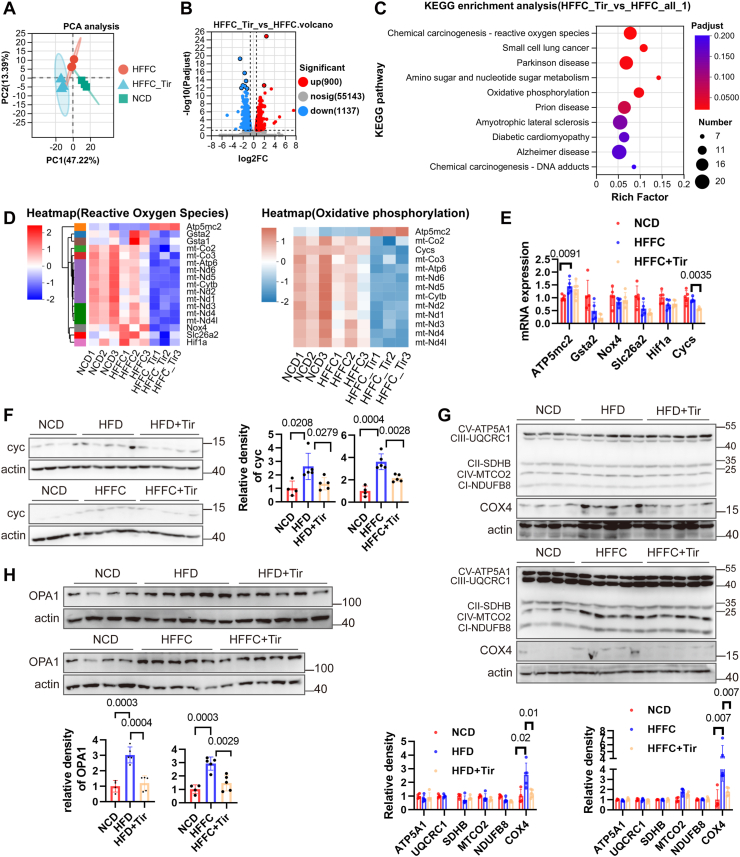
Oxidative phosphorylation was down-regulated after tirzepatide treatment. Transcriptomic sequencing was performed on liver tissues from three groups of mice: the NCD, HFFC, and HFFC with tirzepatide treatment groups. **(A)** Principal component analysis. **(B)** Volcano plot of differential expression. **(C)** KEGG enrichment analysis was performed on the differentially expressed genes between the HFFC group and the HFFC with tirzepatide treatment group. **(D)** Cluster analysis was performed on the genes involved in the reactive oxygen species and oxidative phosphorylation pathways across the NCD, HFFC, and HFFC with tirzepatide treatment groups. **(E)** Quantitative PCR was performed to validate the expression of genes in this pathway, including Atp5mc2, Gsta2, Nox4, Slc26a2, Hif1α, and Cycs. **(F)** Western blot analysis was used to detect Cyc protein expression in liver tissues from the NCD, HFD, HFFC, and HFD or HFFC with tirzepatide treatment groups. **(G)** Western blot analysis was used to detect the protein expression levels of ATP5A1, UQCRC1, SDHB, MTCO2, NDUFB8, and COX4 in liver tissues from the NCD, HFD, HFFC, and HFD or HFFC with tirzepatide treatment groups. **(H)** OPA1 protein expression was analyzed via western blotting. *n* = 5. The data are presented as mean ± SD.

To elucidate the underlying mechanisms by which tirzepatide reduces hepatic lipid accumulation induced by HFD or HFFC diets, we performed transcriptome sequencing. Principal component analysis showed clear separation among the NCD, HFFC, and HFFC with tirzepatide treatment groups, indicating distinct gene expression profiles among them (Fig. 3A). The differential expression volcano plot shows significantly differentially expressed genes among the three groups (Fig. 3B). KEGG enrichment analysis indicated that tirzepatide significantly affected the reactive oxygen species and oxidative phosphorylation pathways (Fig. 3C). Cluster analysis was performed on the genes involved in these two pathways across the NCD, HFFC, and HFFC with tirzepatide treatment groups (Fig. 3D). Subsequent quantitative PCR validation showed that the mRNA levels of Atp5mc2, Gsta2, Nox4, Slc26a2, and Hif1α did not exhibit significant changes, with the exception of Cycs (also known as Cyc), which was down-regulated following tirzepatide treatment (Fig. 3E). Since Cyc is a key protein involved in oxidative phosphorylation, its protein expression was further validated. The results showed that Cyc protein levels were significantly reduced following tirzepatide treatment, consistent with the observed decrease in Cyc mRNA expression (Fig. 3F). In addition to Cyc, the core proteins involved in oxidative phosphorylation are organized into five multi-protein complexes (Complexes I–V), which sequentially transfer electrons through redox reactions to drive the oxidative phosphorylation process. As shown in Figure 3G, ATP5A1 (Complex V), UQCRC1 (Complex III), SDHB (Complex II), MTCO2 (Complex IV), and NDUFB8 (Complex I) showed no significant changes in expression in the livers of tirzepatide-treated mice compared to those in the HFD or HFFC groups. Cytochrome c oxidase 4 (COX4) plays a critical role in the assembly and function of the mitochondrial respiratory chain, influencing the overall activity of the electron transport chain and cellular energy production. As shown in Figure 3G, the HFD or HFFC diet increased COX4 protein expression, whereas tirzepatide treatment reduced it. OPA1 plays a key role in regulating mitochondrial morphology and energy metabolism. As shown in Figure 3H, the HFD or HFFC diet increased OPA1 protein levels, whereas tirzepatide reduced its expression, suggesting a potential inhibitory effect of tirzepatide on mitochondrial oxidative phosphorylation. Taken together, these findings suggest that tirzepatide downregulates mitochondrial oxidative phosphorylation pathways and that tirzapetide may reduce hepatic lipid accumulation through other pathways.

### Tirzapetide reduces hepatic lipid accumulation by down-regulating the expression of CD36 and OBP2A

**Figure 4:**
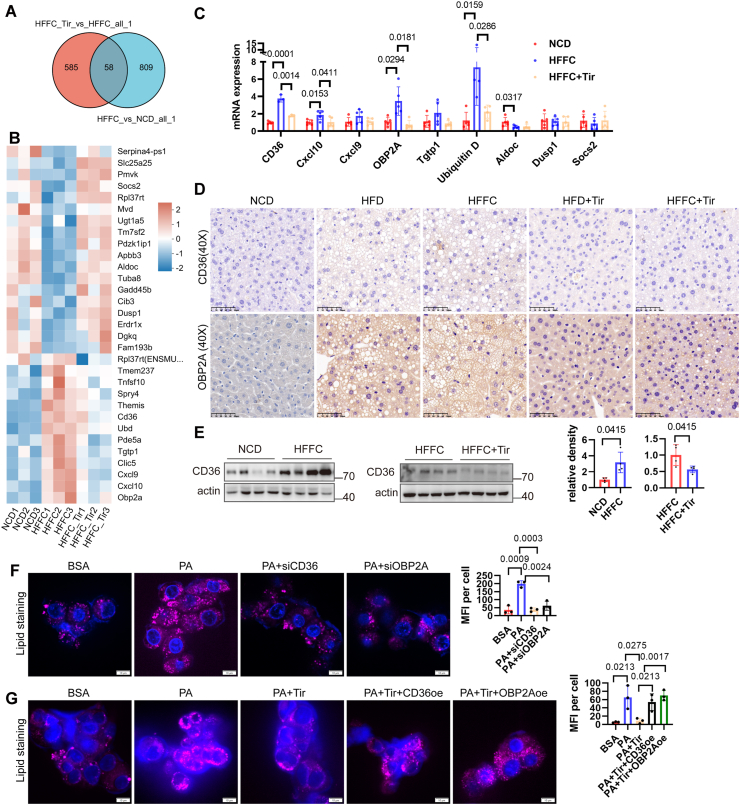
Tirzapetide reduces hepatic lipid accumulation by down-regulating the expression of CD36 and OBP2A. **(A)** Venn analysis of the differentially expressed genes in the livers of mice fed with NCD, HFFC, and HFFC treated with tirzapetide. **(B)** Clustering analysis was performed on overlapping differentially expressed genes with padjust values less than 0.05. **(C)** Quantitative PCR was performed to detect the mRNA expression of specific genes (CD36, Cxcl10, Cxcl9, OBP2A, Tgtp1, Ubiquitin D, Aldoc, Dusp1, Socs2) in the livers of mice fed with NCD, HFFC, and HFFC supplemented with tirzapetide. **(D)** Immunohistochemical staining was performed to detect the protein expression of CD36 and OBP2A in the livers of mice fed with NCD, HFD, HFFC, and HFD or HFFC supplemented with tirzapetide. **(E)** Western blot analysis of CD36 protein expression in the livers of mice fed with NCD, HFFC, and HFFC supplemented with tirzapetide. **(F)** siRNAs targeting CD36 or OBP2A (siCD36 or siOBP2A) were transfected into HepG2 cells for 48 h, followed by treatment with 0.2% BSA or 0.2 mM palmitic acid for the last 24 h. Subsequently, the cells were stained with HCS LipidTOX™ Deep Red neutral lipid stain. **(G)** HepG2 cells were treated for 48 h with 0.2% BSA, 0.2 mM palmitic acid, or palmitic acid combined with 42 nM tirzapetide, with or without transfection of CD36 or OBP2A overexpression plasmids, followed by staining with HCS LipidTOX™ Deep Red neutral lipid stain to assess intracellular lipid accumulation. The average lipid content per cell was quantified using ImageJ software. *n* = 5 for A–E, *n* = 3 for F–G. The data are presented as mean ± SD.

To identify the specific pathways through which tirzapetide reduces hepatic lipid accumulation, we analyzed the differentially expressed genes in the livers of mice fed with NCD, HFFC, or HFFC treated with tirzapetide. Venn analysis identified 58 overlapping differentially expressed genes (Fig. 4A). We selected 30 genes with an adjusted *P* value < 0.05 for hierarchical clustering analysis, as presented in Figure 4B. Based on a literature review, we selected several key metabolism-related genes for further validation using quantitative PCR. The mRNA expression of CD36, OBP2A, Cxcl10, and Ubiquitin D was markedly induced by the HFFC diet, and this induction was effectively suppressed following tirzepatide treatment (Fig. 4C). CD36 and OBP2A facilitate the binding and transport of fatty acids.^20^^,^^21^ Therefore, we validated CD36 and OBP2A protein expression via immunohistochemical staining and western blotting. As shown in Figure 4D and E, the expression of CD36 and OBP2A was increased in both the HFD and HFFC models, which was significantly reduced following tirzepatide treatment. To determine whether tirzepatide affects the intracellular lipid content by regulating the expression of CD36 and OBP2A, we treated HepG2 cells with palmitic acid to induce lipid accumulation and then knocked down or overexpressed CD36 and OBP2A. The results showed that palmitic acid treatment increased the lipid content in HepG2 cells, while knockdown of CD36 or OBP2A significantly reduced cytoplasmic lipid levels (Fig. 4F), suggesting that the expression levels of CD36 and OBP2A influence intracellular lipid accumulation. Tirzepatide was able to reduce palmitic acid-induced lipid accumulation in cells, and this ameliorative effect was blocked by the overexpression of CD36 or OBP2A (Fig. 4G). Taken together, these findings suggest that tirzepatide may reduce hepatic lipid uptake by down-regulating the expression of CD36 and OBP2A in the liver, thereby alleviating intrahepatic lipid accumulation in MASLD.

### Tirzepatide does not affect multiple metabolic pathways in the liver

**Figure 5:**
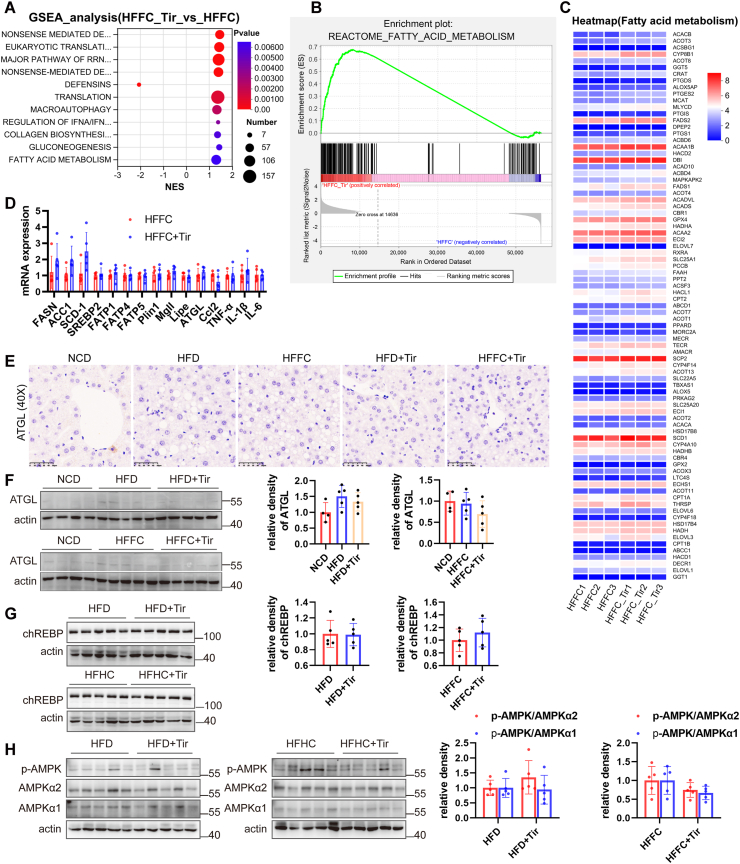
Tirzepatide does not affect multiple metabolic pathways in the liver. **(A)** A gene set enrichment analysis (GSEA) of the differentially expressed genes. **(B)** GSEA focusing on the fatty acid metabolism. **(C)** Enrichment analysis of the fatty acid metabolism gene set. **(D)** Quantitative PCR was used to evaluate the mRNA expression levels of multiple genes involved in fatty acid synthesis, transport, lipolysis, and inflammation in the livers of mice fed with HFFC or HFFC supplemented with tirzapetide. **(E)** The protein expression of adipose triglyceride lipase (ATGL) in the livers of mice fed with NCD, HFD, HFFC, and HFD or HFFC supplemented with tirzapetide was detected by immunohistochemical staining. ATGL **(F)**, chREBP **(G)**, AMPK and p-AMPK (Thr172+Thr183) **(H)** were detected by western blotting. *n* = 5. The data are presented as mean ± SD.

To further explore the effects of tirzepatide on pathways, especially metabolic pathways, we performed a gene set enrichment analysis (GSEA) on the differentially expressed genes. As shown in Figure 5A, the fatty acid metabolism pathway was enriched and modulated by tirzepatide treatment. We performed a GSEA focusing on the fatty acid metabolism pathway. The resulting positive enrichment score indicated that this gene set was predominantly enriched in the HFFC treated with tirzapetide group (Fig. 5B). We further performed enrichment analysis on these 83 genes and found that the expression of fatty acid metabolism-related genes was not significantly altered (Fig. 5C). We further validated the expression of classical metabolism-related genes, and the results showed that tirzepatide treatment had no significant effect on the mRNA expression of genes involved in fatty acid synthesis (SREBP2, FASN, ACC1, and SCD-1), fatty acid transport (FATP4 and FATP5), lipolysis (Mgll, Lipe, and ATGL), or inflammation (TNFα, IL-1β, and IL-6) (Fig. 5D). Additionally, western blotting and immunohistochemical analysis showed low hepatic expression of ATGL protein, with no significant differences observed between the NCD, HFD, HFFC, and tirzepatide-treated groups (Fig. 5E, F). Other key metabolic regulators, such as carbohydrate response element-binding protein (ChREBP) and the energy sensor AMPK, showed no significant activation in response to tirzepatide treatment (Fig. 5G, H). Overall, these findings suggest that tirzepatide has limited effects on multiple metabolic pathways.

### Tirzepatide down-regulated the expression of CD36 and OBP2A while up-regulating ATGL in adipose tissue

**Figure 6:**
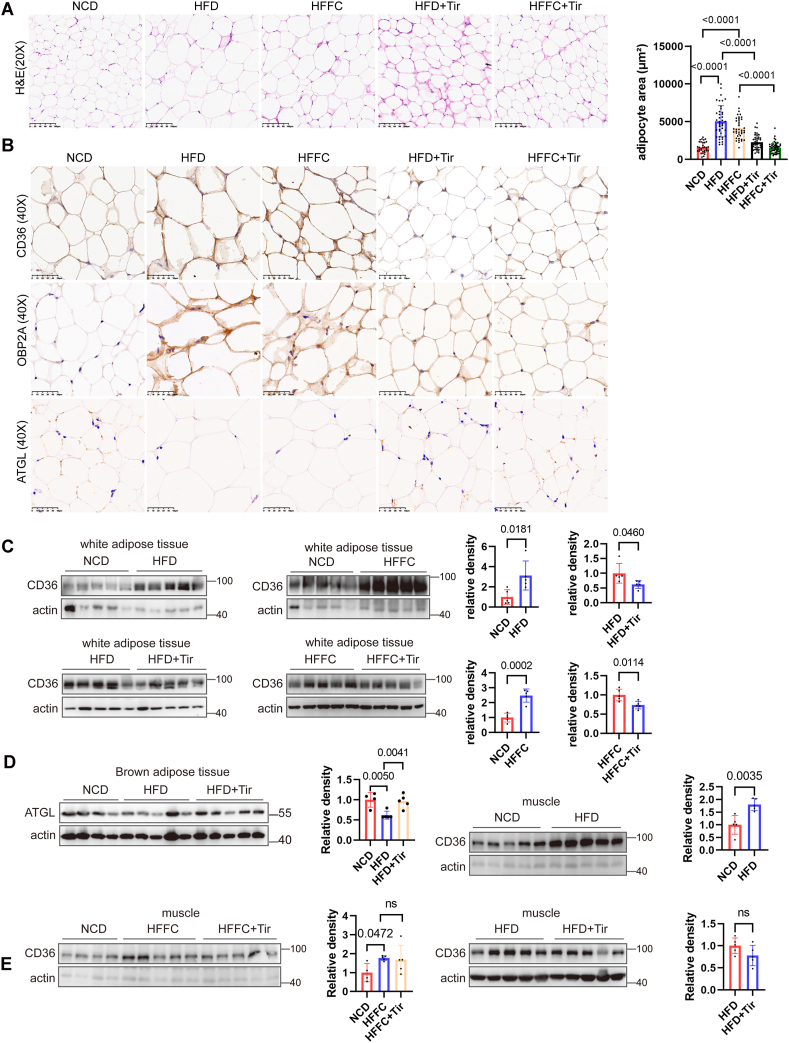
Tirzepatide decreased CD36 and OBP2A expression and increased ATGL expression in adipose tissue. **(A)** HE staining of white adipose tissue from mice fed with NCD, HFD, HFFC, and HFD or HFFC supplemented with tirzapetide. For each group, the areas of at least thirty adipocytes were measured and analyzed statistically. **(B)** Immunohistochemical staining of CD36, OBP2A, and ATGL in white adipose tissue. **(C)** Western blotting analysis of CD36 expression in white adipose tissue. **(D)** ATGL expression in brown adipose tissue was detected via western blotting. **(E)** CD36 protein expression in skeletal muscle tissue was detected via western blotting. *n* = 5. The data are presented as mean ± SD.

Given that metabolic dysregulation in MASLD frequently involves extrahepatic tissues, we assessed the expression of key metabolic proteins in such tissues. HE staining showed significant adipocyte hypertrophy in the HFD and HFFC models, which was notably attenuated by tirzepatide treatment (Fig. 6A), suggesting that tirzepatide may improve obesity. To determine whether the expression of CD36, OBP2A, and ATGL in adipose tissue was consistent with that in liver tissue, we performed immunohistochemical staining. As shown in Figure 6B, CD36 and OBP2A expression in adipose tissue was also elevated in both the HFD and HFFC models, and this increase was significantly attenuated by tirzepatide treatment, consistent with their expression patterns in the liver. ATGL is a key enzyme responsible for regulating lipid storage and mobilization. We observed that ATGL expression in adipose tissue was markedly higher than that in the liver; in both the HFD and HFFC models, ATGL expression in white adipose tissue was significantly reduced, whereas tirzepatide treatment restored ATGL expression (Fig. 6B). Changes in CD36 expression were further confirmed by immunoblotting (Fig. 6C).

Brown adipose tissue is a thermogenic organ involved in energy metabolism and the regulation of obesity. To examine ATGL expression in brown adipose tissue, we performed immunoblot analysis. As shown in Figure 6D, HFD feeding also reduced ATGL protein levels in brown adipose tissue, and tirzepatide treatment restored ATGL expression, suggesting that tirzepatide promotes energy metabolism in adipose tissue by enhancing lipolysis.

CD36 has diverse functions in different tissues.^21^, ^22^, ^23^, ^24^ CD36 directly regulates fatty acid oxidation metabolism in skeletal muscle through mediating the intracellular dynamics of the Fyn-LKB1 complex.^25^ Compared with other fatty acid transport proteins, CD36 may play a more important role in the regulation of fatty acid metabolism in skeletal muscle under conditions of high energy demand, such as exercise.^26^ Therefore, we examined changes in CD36 expression in muscle tissue. As shown in Figure 6E–a high-fat diet enhanced CD36 expression in skeletal muscle, potentially promoting fatty acid β-oxidation; however, tirzepatide treatment did not lead to any further increase in CD36 levels. Taken together, these findings indicate that tirzepatide reduces lipid accumulation in adipose tissue, at least in part, by suppressing CD36 and OBP2A expression and enhancing ATGL expression.

## Discussion

Our study demonstrates that tirzepatide effectively suppresses weight gain, reduces liver weight, markedly improves liver histology, and shows significant therapeutic efficacy in treating MAFLD. In addition to reducing hepatic lipids, tirzepatide demonstrates excellent hypoglycemic effects in MASLD mouse models without causing liver damage. These findings support its potential as a promising therapeutic strategy for MASLD.

ALT and AST are reliable biomarkers of hepatocyte injury. In the steatosis stage of MASLD, ALT and AST levels may remain within the normal range or show only mild elevations. However, as the disease progresses to MASH, hepatic inflammation and hepatocyte damage gradually appear, leading to potentially significant increases in ALT and AST levels.^27^, ^28^, ^29^ Recent studies demonstrated that tirzepatide significantly lowered ALT and AST levels, suggesting its potential to alleviate liver damage.^14^^,^^16^ In this study, after feeding the mice with HFD or HFFC diets for 12 weeks, no significant increase in the serum ALT level was observed, and tirzepatide also showed no significant effect in reducing ALT levels. This may be related to the relatively short duration of the high-fat diet feeding period (12 weeks) and the limited duration of tirzepatide treatment. For AST, 12 weeks of HFD or HFFC feeding induced a mild increase in AST levels, indicating the onset of hepatocyte injury, with a more pronounced effect under the HFFC diet. This may be attributed to the 20 kcal% fructose content in the HFFC diet. Unlike glucose, fructose is a key macronutrient believed to increase the risk of hepatic steatosis and NASH. Excessive fructose consumption can lead to impaired intestinal barrier function and endotoxemia. The resulting endotoxins bind to TLR4, activating hepatic macrophages and inducing inflammation and hepatocyte injury.^30^ Tirzepatide significantly reduced the increase in AST induced by the HFD diet but had no significant ameliorative effect on AST levels in HFFC-fed mice. This difference may also be attributed to the distinct dietary compositions—tirzepatide appears to exert protective effects against hepatocyte injury in MASLD induced by high fat but shows limited efficacy in protecting against hepatocyte damage caused by high fructose. In the future, longer administration periods of tirzepatide are needed, and further research is required to determine whether tirzepatide has protective effects against fructose-induced MASLD pathology.

Mitochondrial oxidation levels in MASLD remain controversial. Previous studies have indicated that patients with MASLD exhibit a greater rate of the tricarboxylic acid cycle activity and enhanced mitochondrial oxidative activity in the liver than healthy individuals. This adaptation may help meet energy demands and prevent excessive lipid accumulation in the early stages of MASLD. However, it can also lead to increased oxidative stress and mitochondrial damage, and ultimately contribute to the progression from simple steatosis to MASH.^31^^,^^32^ Another perspective suggests that there may be no significant difference, or even a reduction, in the rate of mitochondrial fatty acid oxidation in MASLD patients compared to healthy individuals.^33^, ^34^, ^35^ In this study, our enrichment analysis showed that mitochondrial oxidative phosphorylation was significantly affected in MASLD and that the expression of oxidative phosphorylation-related proteins was increased, indicating the up-regulation of energy metabolism to cope with hepatic lipid accumulation. Tirzepatide treatment reduced oxidative phosphorylation, which may reflect an adaptive response to decreased hepatic lipid accumulation. This finding is consistent with a recent study showing that tirzepatide attenuates the up-regulation of fatty acid oxidation-related proteins (such as Cpt1a, Acaa1b, Ehhadh, and Cyp4a10) induced by a high-fat diet.^16^ The reduction in mitochondrial oxidative phosphorylation following tirzepatide treatment may alleviate oxidative stress and decrease lipid peroxidation, thereby delaying the progression of MASLD from steatosis to MASH. However, the reduction in oxidative phosphorylation may lead to impaired energy metabolism, which could be a cause for concern. Our experiments ultimately demonstrated that the main mechanism by which tirzepatide alleviates hepatic lipid accumulation is by downregulating key proteins involved in fatty acid uptake, such as CD36 and OBP2A, thereby reducing lipid intake. Overall, despite the complexity of the regulatory network, tirzepatide still demonstrates significant lipid-lowering effects. In addition, prolonging tirzepatide treatment will allow further investigation into its potential to delay the progression from simple steatosis to MASH through the alleviation of oxidative stress.

Abnormalities in hepatic lipid uptake, *de novo* lipogenesis, fatty acid oxidation, and lipid export, as well as lipid droplet formation and metabolism, can all disrupt hepatic lipid homeostasis.^36^ This study confirms that tirzepatide reduces the expression of CD36 and OBP2A, thereby decreasing lipid uptake, which appears to be the primary mechanism underlying its alleviation of intrahepatic lipid accumulation in MASLD. The regulatory effect of GLP-1 agonists on CD36 expression appears to be significant and consistent.^16^^,^^37^, ^38^, ^39^ For example, semaglutide reduces the expression of CD36, FABP5, ACSL, ACOX3, PLIN2, ANGPTL4, LPL, MGLL, AQP7 and PDK4 involved in lipid metabolism accompanied by decreases in visceral fat accumulation and blood lipids, and improvement in glucose intolerance.^38^^,^^39^ However, our study did not observe significant changes in the expression of other fatty acid transport proteins or cholesterol metabolism-related proteins. This might be due to the relatively short duration of tirzepatide administration. Therefore, in future studies, we will extend the duration of tirzepatide treatment to further observe its effect on liver metabolic homeostasis and the underlying mechanisms and provide a theoretical basis for a comprehensive understanding of tirzepatide.

ATGL influences lipid crosstalk between adipose and liver tissues. The impact of GLP-1 receptor agonists on ATGL expression remains controversial. Su et al reported that liraglutide can alleviate ectopic lipid deposition in the renal tubules of diabetic nephropathy rats by increasing the protein expression levels of ATGL and HSL.^40^ However, other studies have shown that liraglutide does not significantly affect ATGL expression but only reduces the expression of FASN in adipose tissue.^41^ In our study, the expression of ATGL in adipose tissue increased with tirzepatide treatment, leading to increased lipolysis. This may be the reason for the reduced lipid deposition and decreased adipocyte size; however, whether this process causes a surge of free fatty acids into the circulation, which are subsequently taken up by the liver, is worth considering. The role of ATGL in MASLD progression is complex. Previous studies have shown that increased fatty acid flux from adipose tissue to the liver contributes to the development of MASLD and that inhibiting ATGL may attenuate steatohepatitis.^42^, ^43^, ^44^ In this study, we speculate that weekly injections of tirzepatide, while increasing the expression of ATGL in adipose tissue and promoting lipolysis, result in the release of fatty acids into the circulation. However, due to the reduced expression of CD36 and OBP2A in hepatocytes, the influx of fatty acids is not taken up by hepatocytes. Therefore, the enhancement of ATGL expression in adipose tissue by tirzepatide does not mask its direct inhibitory effect on lipid uptake in the liver. Overall, this results in a favorable alleviation of MASLD.

CD36 has distinct functions in the liver, adipose tissue, and muscle. In the liver and adipose tissue, elevated CD36 expression is an unfavorable signal, as it enhances lipid uptake, contributing to obesity and fatty liver disease.^21^ In contrast, in muscle tissue, CD36 is essential for lipid metabolism and the cellular utilization of fatty acids as fuel.^45^ During exercise, CD36 expression in muscle increases to meet heightened energy demands.^46^ In this study, tirzepatide significantly and consistently down-regulated CD36 and OBP2A in both liver and adipose tissues, suggesting that tirzepatide alleviates lipid accumulation in these tissues primarily by reducing lipid uptake, highlighting its therapeutic advantages. However, tirzepatide did not have a significant effect on CD36 expression in muscle tissue, suggesting that its ability to increase energy expenditure is limited and that its lipid-lowering effects are instead achieved by targeting lipid uptake in specific organs, such as the liver and adipose tissue. Therefore, the differential regulation of CD36 by tirzepatide across tissues provides strong evidence for its favorable targeting ability in the treatment of MASLD and obesity.

In summary, the multi-faceted metabolic regulatory effects of tirzepatide in MASLD strongly support its potential as a novel therapeutic approach for metabolic diseases. Future studies may need to explore the long-term effects and mechanisms of tirzepatide to fully understand its benefits and optimize its application in treating MASLD.

## CRediT authorship contribution statement

**Yun Li:** Funding acquisition, Data curation, Software, Methodology, Writing – original draft, Conceptualization. **Wencong Sun:** Investigation, Data curation, Methodology. **Hong Liu:** Investigation, Methodology, Data curation. **Xiong Z. Ruan:** Supervision, Writing – review & editing.

## Funding

This work was supported by the National Key R&D Program of China (No. 2022YFC2502501); the 10.13039/501100001809National Natural Science Foundation of China (No. 32030054, U23A20415, 82000539), the Chongqing Natural Science Foundation General Program (China) (No. CSTB2024NSCQ-MSX0219), the Young Top Talent Program for Medicine in Chongqing, China (No. YXQN202440), and the Fourth Batch of Kuanren Elite Outstanding Youth Talent Project (China) (No. 202417-42).

## Conflict of interests

There are no competing interests for any author.

## References

[1] Younossi Z.M., Golabi P., Paik J.M., Henry A., Van Dongen C., Henry L. (2023). The global epidemiology of nonalcoholic fatty liver disease (NAFLD) and nonalcoholic steatohepatitis (NASH): a systematic review. Hepatology.

[2] Harrison S.A., Allen A.M., Dubourg J., Noureddin M., Alkhouri N. (2023). Challenges and opportunities in NASH drug development. Nat Med.

[3] Loomba R., Abraham M., Unalp A. (2012). Association between diabetes, family history of diabetes, and risk of nonalcoholic steatohepatitis and fibrosis. Hepatology.

[4] Quek J., Chan K.E., Wong Z.Y. (2023). Global prevalence of non-alcoholic fatty liver disease and non-alcoholic steatohepatitis in the overweight and obese population: a systematic review and meta-analysis. Lancet Gastroenterol Hepatol.

[5] El K., Douros J.D., Willard F.S. (2023). The incretin co-agonist tirzepatide requires GIPR for hormone secretion from human islets. Nat Metab.

[6] Frías J.P., Davies M.J., Rosenstock J. (2021). Tirzepatide versus semaglutide once weekly in patients with type 2 diabetes. N Engl J Med.

[7] Rosenstock J., Wysham C., Frías J.P. (2021). Efficacy and safety of a novel dual GIP and GLP-1 receptor agonist tirzepatide in patients with type 2 diabetes (SURPASS-1): a double-blind, randomised, phase 3 trial. Lancet.

[8] Rodriguez P.J., Goodwin Cartwright B.M., Gratzl S. (2024). Semaglutide vs tirzepatide for weight loss in adults with overweight or obesity. JAMA Intern Med.

[9] Battelino T., Bergenstal R.M., Rodríguez A. (2022). Efficacy of once-weekly tirzepatide versus once-daily insulin degludec on glycaemic control measured by continuous glucose monitoring in adults with type 2 diabetes (SURPASS-3 CGM): a substudy of the randomised, open-label, parallel-group, phase 3 SURPASS-3 trial. Lancet Diabetes Endocrinol.

[10] Ludvik B., Giorgino F., Jódar E. (2021). Once-weekly tirzepatide versus once-daily insulin degludec as add-on to metformin with or without SGLT2 inhibitors in patients with type 2 diabetes (SURPASS-3): a randomised, open-label, parallel-group, phase 3 trial. Lancet.

[11] Malhotra A., Grunstein R.R., Fietze I. (2024). Tirzepatide for the treatment of obstructive sleep apnea and obesity. N Engl J Med.

[12] Zhao L., Cheng Z., Lu Y. (2024). Tirzepatide for weight reduction in Chinese adults with obesity: the SURMOUNT-CN randomized clinical trial [published correction appears in JAMA. 2024 Aug 20;332(7):595.]. JAMA.

[13] Aronne L.J., Sattar N., Horn D.B. (2024). Continued treatment with tirzepatide for maintenance of weight reduction in adults with obesity: the SURMOUNT-4 randomized clinical trial. JAMA.

[14] Hartman M.L., Sanyal A.J., Loomba R. (2020). Effects of novel dual GIP and GLP-1 receptor agonist tirzepatide on biomarkers of nonalcoholic steatohepatitis in patients with type 2 diabetes. Diabetes Care.

[15] Valenzuela-Vallejo L., Guatibonza-García V., Mantzoros C.S. (2022). Recent guidelines for non-alcoholic fatty liver disease (NAFLD)/fatty liver disease (FLD): are they already outdated and in need of supplementation?. Metabolism.

[16] Liang J., Liu H., Lv G. (2025). Exploring the molecular mechanisms of tirzepatide in alleviating metabolic dysfunction-associated fatty liver in mice through integration of metabolomics, lipidomics, and proteomics. Lipids Health Dis.

[17] Loomba R., Hartman M.L., Lawitz E.J. (2024). Tirzepatide for metabolic dysfunction-associated steatohepatitis with liver fibrosis. N Engl J Med.

[18] Friedman S.L., Neuschwander-Tetri B.A., Rinella M., Sanyal A.J. (2018). Mechanisms of NAFLD development and therapeutic strategies. Nat Med.

[19] Chalasani N., Younossi Z., Lavine J.E. (2018). The diagnosis and management of nonalcoholic fatty liver disease: practice guidance from the American association for the study of liver diseases. Hepatology.

[20] Ekim Kocabey A., Schneiter R. (2024). Human lipocalins bind and export fatty acids through the secretory pathway of yeast cells. Front Microbiol.

[21] Li Y., Yang P., Zhao L. (2019). CD36 plays a negative role in the regulation of lipophagy in hepatocytes through an AMPK-dependent pathway. J Lipid Res.

[22] Yang X., Okamura D.M., Lu X. (2017). CD36 in chronic kidney disease: novel insights and therapeutic opportunities. Nat Rev Nephrol.

[23] Son N.H., Basu D., Samovski D. (2018). Endothelial cell CD36 optimizes tissue fatty acid uptake. J Clin Investig.

[24] Ma Y., Huang L., Zhang Z. (2023). CD36 promotes tubular ferroptosis by regulating the ubiquitination of FSP1 in acute kidney injury. Genes Dis.

[25] Samovski D., Sun J., Pietka T. (2015). Regulation of AMPK activation by CD36 links fatty acid uptake to β-oxidation. Diabetes.

[26] Holloway G.P., Luiken J.P., Glatz J.C., Spriet L.L., Bonen A. (2008). Contribution of FAT/CD36 to the regulation of skeletal muscle fatty acid oxidation: an overview. Acta Physiol.

[27] Fracanzani A.L., Valenti L., Bugianesi E. (2008). Risk of severe liver disease in nonalcoholic fatty liver disease with normal aminotransferase levels: a role for insulin resistance and diabetes. Hepatology.

[28] Loomba R., Amangurbanova M., Bettencourt R. (2024). MASH resolution index: development and validation of a non-invasive score to detect histological resolution of MASH. Gut.

[29] Shankar S.S., Daniels S.J., Robertson D. (2024). Safety and efficacy of novel incretin co-agonist cotadutide in biopsy-proven noncirrhotic MASH with fibrosis. Clin Gastroenterol Hepatol.

[30] Todoric J., Di Caro G., Reibe S. (2020). Fructose stimulated *de novo* lipogenesis is promoted by inflammation. Nat Metab.

[31] Sunny N.E., Parks E.J., Browning J.D., Burgess S.C. (2011). Excessive hepatic mitochondrial TCA cycle and gluconeogenesis in humans with nonalcoholic fatty liver disease. Cell Metab.

[32] Koliaki C., Szendroedi J., Kaul K. (2015). Adaptation of hepatic mitochondrial function in humans with non-alcoholic fatty liver is lost in steatohepatitis. Cell Metab.

[33] Zhang D., Zhao Y., Zhang G. (2024). Suppression of hepatic ChREBP⍺-CYP2C50 axis-driven fatty acid oxidation sensitizes mice to diet-induced MASLD/MASH. Mol Metabol.

[34] Petersen K.F., Dufour S., Mehal W.Z., Shulman G.I. (2024). Glucagon promotes increased hepatic mitochondrial oxidation and pyruvate carboxylase flux in humans with fatty liver disease. Cell Metab.

[35] Petersen K.F., Befroy D.E., Dufour S., Rothman D.L., Shulman G.I. (2016). Assessment of hepatic mitochondrial oxidation and pyruvate cycling in NAFLD by (13)C magnetic resonance spectroscopy. Cell Metab.

[36] Jeon S., Carr R. (2020). Alcohol effects on hepatic lipid metabolism. J Lipid Res.

[37] Dai Y., Dai D., Wang X., Ding Z., Li C., Mehta J.L. (2014). GLP-1 agonists inhibit ox-LDL uptake in macrophages by activating protein kinase A. J Cardiovasc Pharmacol.

[38] Kong M.W., Gao Y., Xie Y.Y. (2022). Mechanism of GLP-1 receptor agonists-mediated attenuation of palmitic acid-induced lipotoxicity in L6 myoblasts. BioMed Res Int.

[39] Zhu R., Chen S. (2023). Proteomic analysis reveals semaglutide impacts lipogenic protein expression in epididymal adipose tissue of obese mice. Front Endocrinol.

[40] Su K., Yi B., Yao B.Q. (2020). Liraglutide attenuates renal tubular ectopic lipid deposition in rats with diabetic nephropathy by inhibiting lipid synthesis and promoting lipolysis. Pharmacol Res.

[41] Chen J., Zhao H., Ma X. (2017). GLP-1/GLP-1R signaling in regulation of adipocyte differentiation and lipogenesis. Cell Physiol Biochem.

[42] Schreiber R., Xie H., Schweiger M. (2019). Of mice and men: the physiological role of adipose triglyceride lipase (ATGL). Biochim Biophys Acta Mol Cell Biol Lipids.

[43] Fuchs C.D., Radun R., Dixon E.D. (2022). Hepatocyte-specific deletion of adipose triglyceride lipase (adipose triglyceride lipase/patatin-like phospholipase domain containing 2) ameliorates dietary induced steatohepatitis in mice. Hepatology.

[44] Dixon E.D., Claudel T., Nardo A.D. (2025). Inhibition of ATGL alleviates MASH *via* impaired PPARα signalling that favours hydrophilic bile acid composition in mice. J Hepatol.

[45] Glatz J.F.C., Heather L.C., Luiken J.J.F.P. (2024). CD36 as a gatekeeper of myocardial lipid metabolism and therapeutic target for metabolic disease. Physiol Rev.

[46] Ramos-Jiménez A., Zavala-Lira R.A., Moreno-Brito V., González-Rodríguez E. (2022). FAT/CD36 participation in human skeletal muscle lipid metabolism: a systematic review. J Clin Med.

